# Maternal thyroid hormone insufficiency during pregnancy and risk of neurodevelopmental disorders in offspring: A systematic review and meta‐analysis

**DOI:** 10.1111/cen.13550

**Published:** 2018-02-08

**Authors:** William Thompson, Ginny Russell, Genevieve Baragwanath, Justin Matthews, Bijay Vaidya, Jo Thompson‐Coon

**Affiliations:** ^1^ NIHR Collaboration for Leadership in Applied Health Research and Care South West Peninsula (PenCLAHRC) University of Exeter Medical School University of Exeter Exeter UK; ^2^ Centre for Study of the Life Sciences University of Exeter Medical School University of Exeter Exeter UK; ^3^ Department of Endocrinology Royal Devon & Exeter Hospital NHS Trust Exeter UK; ^4^ Institute of Health Research University of Exeter Medical School University of Exeter Exeter UK; ^5^ Department of Endocrinology University of Exeter Medical School University of Exeter Exeter UK

**Keywords:** autism, hypothyroxinaemia, intelligent quotient, pregnancy, subclinical hypothyroidism, thyroid

## Abstract

**Background:**

In the last 2 decades, several studies have examined the association between maternal thyroid hormone insufficiency during pregnancy and neurodevelopmental disorders in children and shown conflicting results.

**Aim:**

This systematic review aimed to assess the evidence for an association between maternal thyroid hormone insufficiency during pregnancy and neurodevelopmental disorders in children. We also sought to assess whether levothyroxine treatment for maternal thyroid hormone insufficiency improves child neurodevelopment outcomes.

**Methods:**

We performed systematic literature searches in MEDLINE, EMBASE, PSYCinfo, CINAHL, AMED, BNI, Cochrane, Scopus, Web of Science, GreyLit, Grey Source and Open Grey (latest search: March 2017). We also conducted targeted web searching and performed forwards and backwards citation chasing. Meta‐analyses of eligible studies were carried out using the random‐effects model.

**Results:**

We identified 39 eligible articles (37 observational studies and 2 randomized controlled trials [RCT]). Meta‐analysis showed that maternal subclinical hypothyroidism and hypothyroxinaemia are associated with indicators of intellectual disability in offspring (odds ratio [OR] 2.14, 95% confidence interval [CI] 1.20 to 3.83, *P* = .01, and OR 1.63, 95% CI 1.03 to 2.56, *P* = .04, respectively). Maternal subclinical hypothyroidism and hypothyroxinaemia were not associated with attention deficit hyperactivity disorder, and their effect on the risk of autism in offspring was unclear. Meta‐analysis of RCTs showed no evidence that levothyroxine treatment for maternal hypothyroxinaemia or subclinical hypothyroidism reduces the incidence of low intelligence quotient in offspring.

**Limitations:**

Although studies were generally of good quality, there was evidence of heterogeneity between the included observational studies (*I* ^2^ 72%‐79%).

**Conclusion:**

Maternal hypothyroxinaemia and subclinical hypothyroidism may be associated with intellectual disability in offspring. Currently, there is no evidence that levothyroxine treatment, when initiated 8‐ to 20‐week gestation (mostly between 12 and 17 weeks), for mild maternal thyroid hormone insufficiency during pregnancy reduces intellectual disability in offspring.

## 1 INTRODUCTION

Thyroid hormone is essential for optimum neurological development of the foetus. However, the foetal thyroid gland is not functional until the 12‐14th week of gestation,^1^ and during that period, the foetus is solely dependent on thyroxine from the mother. Therefore, it is plausible that maternal thyroid hormone insufficiency, particularly in early pregnancy, could impair foetal neurodevelopment. Indeed, in recent years, several studies have shown that even mild maternal thyroid hormone insufficiency (including subclinical hypothyroidism and isolated hypothyroxinaemia) during pregnancy is associated with various types of neurodevelopmental disorders in children, including reduced intelligence quotient (IQ) scores,^2^ autism^3^ and attention deficit hyperactivity disorder (ADHD).^4^ However, other studies have shown conflicting results,^5^, ^6^ and there remains an uncertainty whether levothyroxine treatment in mild maternal thyroid hormone insufficiency improves neurodevelopmental outcomes in children.^7^ These observations have led to an ongoing debate about whether all pregnant women should be screened and treated for thyroid dysfunction.

We therefore conducted a systematic review and meta‐analysis to assess the evidence for an association between maternal thyroid hormone insufficiency during pregnancy and neurodevelopmental disorders in childhood, with a focus on intellectual disability, autism spectrum disorders and ADHD, which show substantial comorbidity.^8^ We also assessed the evidence from randomized controlled trials (RCTs) of the effects of levothyroxine treatment in pregnant mothers with thyroid hormone insufficiency on child neurodevelopmental outcomes.

## 2 METHODS

A systematic review was conducted according to the best practice guidelines recommended by the Centre for Research Dissemination^9^ and is reported in accordance with the PRISMA reporting guidelines.^10^ A predefined protocol was developed and is registered with PROSPERO (2016: CRD42016032790).

### 2.1 Literature search

A comprehensive search syntax using MeSH and free text terms was developed for MEDLINE (Appendix S1) and adapted as appropriate for the other searched databases: EMBASE, PsycInfo, CINAHL, AMED, the British Nursing Index, the Cochrane Central Register of Controlled Trials, Scopus, Web of Science, GreyLit, Grey Source and Open Grey (WT). MEDLINE, EMBASE and PsychInfo were searched using OVID; CINAHL and AMED were searched using EBSCOhost; the British Nursing Index was searched using ProQuest; the Cochrane central Register of Controlled Trials was searched using Wiley; Scopus was searched using Elsevier; and Web of Science was searched using Thomson‐Reuters platforms. All databases were searched from inception to February 2016, with an update search conducted in March 2017. No study design or language restrictions were imposed, but case‐reports, case‐series, reviews, editorials and commentaries were excluded.

The search strategy consisted of 3 blocks of terms, those for (i) hypothyroidism, (ii) neurodevelopmental disorders and (iii) mothers and pregnancy. Terms for neurodevelopmental disorders were taken from the 4th and 5th edition of the Diagnostic and Statistical Manual for Mental Disorders (DSM‐IV and DSM5)^11^ with additional terminology from the International Classifications of Diseases 10th edition (ICD‐10).^12^

The “Research” section of the websites of the following organizations was reviewed (March 2016) in detail for additional publications: Autistica, Autism Speaks, Dame Shirley Foundation, International Society for Autism Research, Simons Foundation, Waterloo foundation, National Autistic Society, British Thyroid Foundation, British Thyroid Association, Society for Endocrinology and the American Thyroid Association. Forwards (using Scopus) and backwards citation searching was used to identify additional relevant papers.

### 2.2 Eligibility criteria

Observational studies were included if they investigated the association of maternal thyroid hormone insufficiency (including overt hypothyroidism, subclinical hypothyroidism and maternal hypothyroxinaemia) in mothers during pregnancy with neurodevelopmental outcomes in their children. Overt hypothyroidism was defined as high serum thyroid‐stimulating hormone (TSH) levels and low‐serum‐free thyroxine (fT4) or total thyroxine (tT4)_,_ subclinical hypothyroidism as high TSH levels with normal fT4 or tT4 levels and hypothyroxinaemia as normal TSH with low fT4 or tT4 levels. Studies measuring TSH, fT4 and tT4 as continuous variables were also included. Studies were classified as indicating intellectual disability where IQ, language delay or global developmental delay was measured, and were classified as ADHD or autism studies where a diagnosis or a validated scale for measuring these conditions were reported as an outcome measure in the offspring. We excluded studies that focussed on psychomotor development rather than neuropsychological development. Studies were included if the children studied were between 0 and 18 years old. RCTs were included if they studied pregnant women with thyroid hormone insufficiency, and randomly assigned them to treatment with levothyroxine or to a control group and reported children's neurodevelopmental outcomes.

Only studies published after 1994 were included in the analysis, coinciding with the publication of DSM‐IV which introduced changes in terminology used to describe neurodevelopmental outcomes.

### 2.3 Study selection

The search results were uploaded to reference management software (Endnote X7). Titles and abstracts were screened for relevance independently by 2 reviewers (WT and JTC or GB), with any disagreements being resolved by discussion and involvement of a third reviewer (JTC or GB) where necessary. The full text of potentially relevant papers was retrieved and screened in the same way using the prespecified inclusion and exclusion criteria. All duplicate papers were double‐checked and excluded. “Sibling” papers derived from the same parent study were identified and linked.

### 2.4 Data collection

For each study, data on study and participant characteristics, intervention/exposure, thyroid status and relevant neurodevelopmental outcomes were extracted by 1 reviewer (WT) into a bespoke data extraction form, which was piloted on a random sample of studies and refined. All data extraction was checked by a second reviewer (JTC or GB) with discrepancies resolved by discussion and involvement of a third reviewer (JTC or GB) where necessary. Authors were contacted to provide clarification or additional data if necessary.

### 2.5 Quality assessment

The quality of the design and reporting of included studies was assessed using the Downs and Black checklist^13^ by 1 reviewer (WT) and checked by a second (JTC or GB), a reliable and valid quality index for the appraisal of both RCTs and nonrandomized studies. Discrepancies were resolved through discussion and involvement of a third reviewer (JTC or GB) where necessary.

### 2.6 Data analysis

Studies were tabulated and described narratively in the first instance. Studies were only included in the meta‐analysis with the following criteria: (i) they used a fixed predictor, (ii) that the fixed predictor used was at a threshold of clinical relevance, (iii) that they provided numerical data and (iv) used an outcome assessment that was well known and validated. Where meta‐analysis was possible, the following prespecified principles were applied to the data prior to analysis: (i) for studies using multiple tests for the same outcome, the most commonly used test was reported, (ii) where available, the total test score was used rather than subscales, (iii) where a trait was measured over multiple time periods, the latest point of measuring would be chosen, as neurodevelopmental traits at an older age tend to be more stable, (iv) where multiple cut‐offs of TSH and fT4 were measured, the most extreme cut‐off was used and when an outcome was measured both against a continuous thyroid hormone measure and against a cut‐off value (eg cut‐off of the 10th percentile fT4 for hypothyroxinaemia) the cut‐off value was used, (v) where thyroid hormone samples were collected at multiple times in pregnancy, the earliest time was chosen as hypothyroidism in early pregnancy is believed to be more problematic than in later pregnancy, (vi) where both fT4 and tT4 were measured, the value for fT4 was used and (vii) adjusted measures were used when provided.

Where possible, continuous results including those derived from regression coefficients were converted to odds ratios using the method described by Chinn and colleagues.^14^, ^15^ Continuous results were also converted to odds ratios using the method described by Suissa^16^ and Whitehead^17^ and explored the impact of the conversion method used in sensitivity analyses. Meta‐analyses were also performed using studies only reporting odds ratios. We also carried out sensitivity analysis (post hoc) by carrying out meta‐analyses where the results were split by the point in gestation when maternal thyroid hormones were measured, to test the potential adverse effects of thyroid dysfunction in early pregnancy vs late pregnancy.

Some studies gave results that were split by a covariate, which were not comparable with other studies. Data from the study reported by Päkkilä et al^18^ for ADHD, for example, were split by gender, hence the authors were contacted for results for both genders combined.

Meta‐analysis was conducted using STATA using the random‐effects model.

## 3 RESULTS

### 3.1 Search results

**Figure 1:**
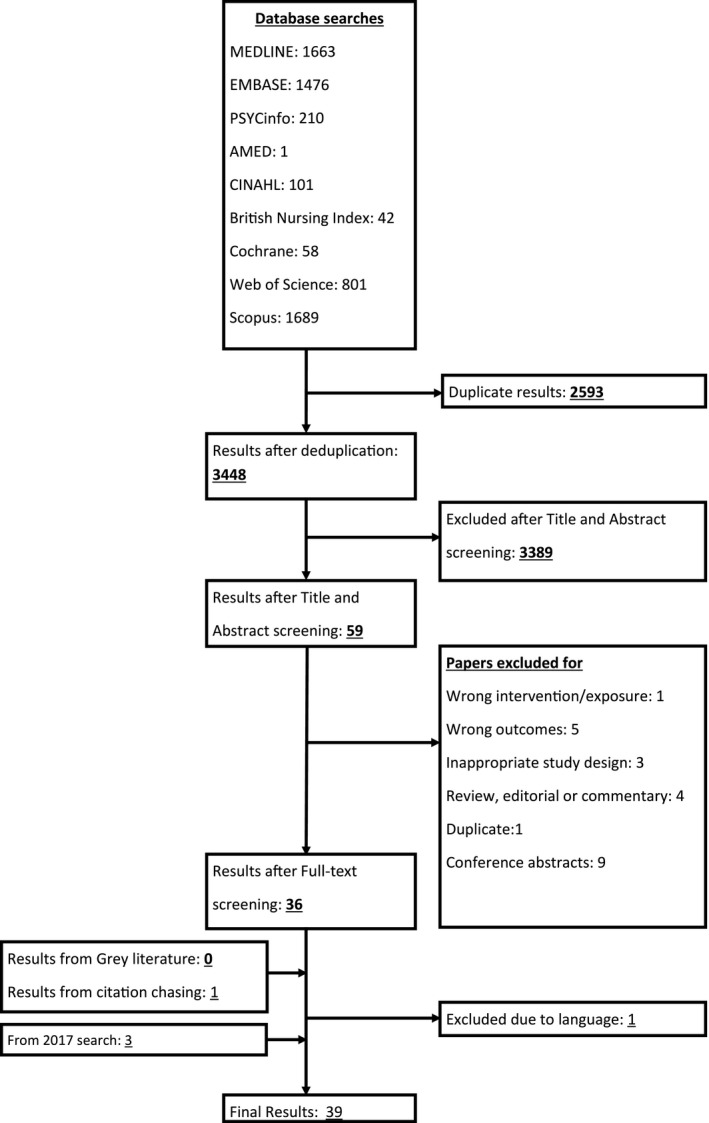
The PRISMA diagram showing search strategy and exclusion criteria at each step

In total, 39 original articles were found to be eligible for this systematic review, with 37 observational studies and 2 RCTs^2^, ^3^, ^5^, ^6^, ^7^, ^18^, ^19^, ^20^, ^21^, ^22^, ^23^, ^24^, ^25^, ^26^, ^27^, ^28^, ^29^, ^30^, ^31^, ^32^, ^33^, ^34^, ^35^, ^36^, ^37^, ^38^, ^39^, ^40^, ^41^, ^42^, ^43^, ^44^, ^45^, ^46^, ^47^, ^48^, ^49^, ^50^, ^51^ (Table S1). Overall 909 176 people participated in these studies (857 014 of them being from one study^37^). A flow diagram summarizing search results is shown in Figure 1.

### 3.2 Risk of bias and heterogeneity

Observational studies have an inherent risk of selection bias and confounding. Quality assessment using the Downs and Black checklist^13^ (Table S2) showed that some of these studies also had noticeable risk of bias relating to attrition bias (Table S3). One study had a high risk of bias, as it had inadequate information on participant demographics.^23^ One of the RCTs^7^ was not placebo‐controlled and suffered from a high attrition rate (Table S4).

There was variation between the included studies in terms of timing of the assessment of thyroid function during pregnancy, definitions of thyroid dysfunction and child age at the time of assessment of neurodevelopment outcome measures (Table S1).

### 3.3 Observational studies excluded from meta‐analysis

In total, 26 studies (24 observational studies and both RCTs) were included in the meta‐analysis. Five studies presenting regression analyses were excluded^26^, ^29^, ^30^, ^35^, ^45^ because they did not provide regression information in a form that would allow straightforward conversion into odds ratios: for example, 2 studies^29^, ^30^ provided linear regression results with continuous not binary predictors. A further 8 articles were excluded from the meta‐analysis for the following reasons: sibling papers with no additional data (n = 2),^21^, ^51^ lacking numerical data (n = 1),^44^ using a novel outcome measure that was not validated or suitable for meta‐analysis (n = 1),^34^ reporting odds ratios (using logistic regression) but using continuous predictors (n = 1)^39^ and reporting psychomotor outcomes only (n = 3).^19^, ^24^, ^42^ Details on why studies were excluded from the meta‐analysis are shown in Table S5.

### 3.4 Association between maternal thyroid hormone insufficiency and indicators of intellectual disability

#### 3.4.1 Overt hypothyroidism

There were inadequate data to perform a meta‐analysis on the association between overt hypothyroidism and indicators of intellectual disability. Two studies claimed to measure overt hypothyroidism,^2^, ^48^ but in practice, the exposed groups in these studies consisted of a mixture of overt and subclinical hypothyroidism cases as the hypothyroidism diagnosis was based entirely on TSH levels rather than TSH and fT_4_ combined.

#### 3.4.2 Subclinical hypothyroidism

**Figure 2:**
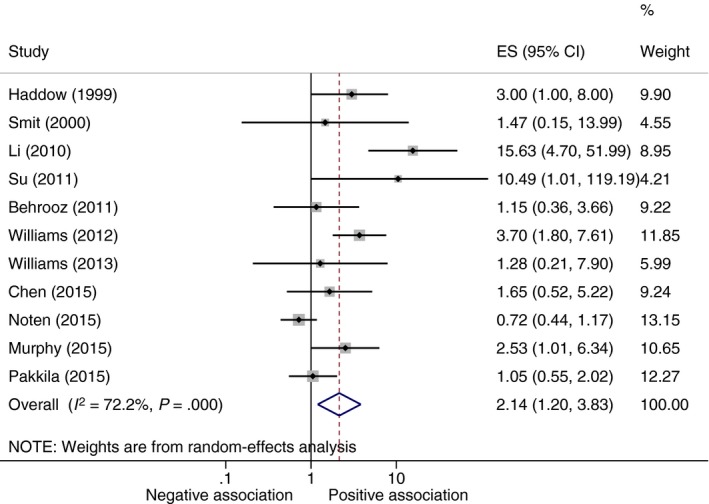
Meta‐analysis of studies on association between maternal subclinical hypothyroidism and indicators of intellectual disability in offspring. ES, Odds ratio point estimate; negative association, trait associated with decreased odds of neurodevelopmental impairment; positive association, trait associated with increased odds of neurodevelopmental impairment

Eleven studies were included in the meta‐analysis (Figure 2). There was evidence of an effect of maternal subclinical hypothyroidism on the risk of intellectual impairment in children (odds ratio [OR] 2.14, 95% confidence interval [CI] 1.20 to 3.83, *P* = .01). Haddow et al^2^ and Päkkilä et al^48^ had some cases of overt hypothyroidism within the exposed group, though removal of these studies made no difference to the significance of the result (OR 2.33, 95% CI 1.12 to 4.87, *P* = .02) (Figure S1). When only studies reporting odds ratios directly (6 studies) were included in the meta‐analysis, maternal subclinical hypothyroidism was not significantly associated with indicators of intellectual disability, though the magnitude of the point estimate was similar (OR 2.37, 95% CI 0.96 to 5.85) (Table S6). When only studies (n = 4) that measured TSH before 12 weeks were included in the meta‐analysis, maternal subclinical hypothyroidism was not significantly associated with indicators of intellectual disability, and the magnitude of the point estimate was reduced (OR 1.11, 95% CI 0.66 to 1.88, *P* = .7) (Figure S5). The 5 studies are not included in the subclinical hypothyroidism and risk of intellectual impairment meta‐analysis^26^, ^27^, ^29^, ^35^, ^39^ found no association between maternal subclinical hypothyroidism and impaired neuropsychological development in children.

#### 3.4.3 Hypothyroxinaemia

**Figure 3:**
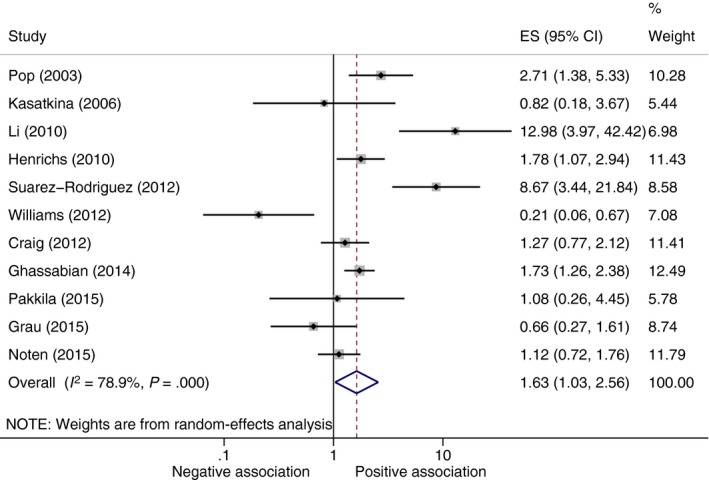
Meta‐analysis of studies on association between maternal hypothyroxinaemia and indicators of intellectual disability in offspring. ES, Odds ratio point estimate; negative association, trait associated with decreased odds of neurodevelopmental impairment; positive association, trait associated with increased odds of neurodevelopmental impairment

Eleven studies were included in the meta‐analysis (Figure 3). Compared to those children born to euthyroid mothers, children born to mothers with hypothyroxinaemia were significantly more likely to show signs of intellectual impairment (OR 1.63, 95% CI 1.03 to 2.56, *P* = .04). The 3 studies excluded from the hypothyroxinaemia and risk of intellectual impairment meta‐analysis^26^, ^29^, ^35^ found no association between maternal hypothyroxinaemia and impaired neurodevelopment in children. Our sensitivity analyses showed that the method used to convert continuous measures to odds ratios had little impact on interpretation (Table S6). When only studies reporting odds ratios (4 studies) were included in the meta‐analysis, maternal hypothyroxinaemia was not significantly associated with indicators of intellectual disability, though the magnitude of the point estimate was similar (OR 2.11, 95% CI 0.92 to 4.83) (Table S6). When only studies (n = 4) that measured fT_4_ before 12 weeks were included in the meta‐analysis, maternal hypothyroxinaemia was not significantly associated with indicators of intellectual disability, though the magnitude of the point estimate was similar (OR 1.22, 95% CI 0.55 to 2.74, *P* = .62) (Figure S6).

### 3.5 Association between maternal thyroid hormone insufficiency and autism

Three studies investigated the link between maternal overt hypothyroidism and autism, 3 studies investigated the link between maternal subclinical hypothyroidism and autism, and 2 studies investigated the link between maternal hypothyroxinaemia and autism. Of the 3 studies that investigated the link between maternal overt hypothyroidism and autism, 2 studies^37^, ^50^ are based on overt hypothyroidism status on hospital records, whilst one study^40^ measured the mother's thyroid hormones directly. As there was no information on thyroid hormone levels on the mothers with hypothyroidism based on hospital records,^37^, ^50^ we analysed the results from these studies separately from the study with the direct thyroid hormone measurement in participants.^40^ There was no statistically significant effect of maternal overt hypothyroidism based on hospital records on autism in children (OR 2.12, 95% CI 0.75 to 6.00, *P* = .12) (Figure S2). The one study that measured thyroid hormones directly^40^ found no association between maternal overt hypothyroidism and autism. Of the 3 studies that investigated the link between maternal subclinical hypothyroidism and autism,^3^, ^39^, ^40^ 2 used continuous predictors^3^, ^39^ and were excluded from meta‐analysis, thus no meta‐analysis could take place. None of the 3 studies^3^, ^39^, ^40^ found a positive association between maternal subclinical hypothyroidism and autism. Of the 2 studies that investigated the association between maternal hypothyroxinaemia and autism,^3^, ^40^ one used a continuous predictor^40^ thus was excluded, thus no meta‐analysis could take place. The study that used a continuous predictor^40^ found no association between maternal hypothyroxinaemia and autism, whilst the one that used a binary predictor^3^ did. Therefore, the effect of maternal thyroid hormone insufficiency on the risk of autism in the offspring is unclear.

### 3.6 Association between maternal hypothyroidism and ADHD

One study investigated the link between maternal overt hypothyroidism and ADHD, 5 studies investigated the link between maternal subclinical hypothyroidism and ADHD (2 were included in the meta‐analysis), and 5 studies investigated the link between maternal hypothyroxinaemia and ADHD (2 were included in the meta‐analysis). The one study that investigated the link between maternal overt hypothyroidism and ADHD in children found no significant association (hazard ratio 1.10, 95% CI 0.98 to 1.25).^37^ Our meta‐analysis found no association between maternal subclinical hypothyroidism and ADHD in children (OR 1.58, 95% CI 0.5 to 5.0, *P* = .44) (Figure S3) or between maternal hypothyroxinaemia and ADHD in children (OR 1.34, 95% CI 0.17 to 10.47, *P* = .78) (Figure S4). Of the 3 studies not included in meta‐analysis, 2 studies^29^, ^45^ found no association between either subclinical hypothyroidism or hypothyroxinaemia and ADHD, whilst one study^30^ found an association between high TSH and externalizing symptoms.

### 3.7 Effect of levothyroxine treatment in maternal thyroid hormone insufficiency on neurodevelopment outcomes in children

**Figure 4:**
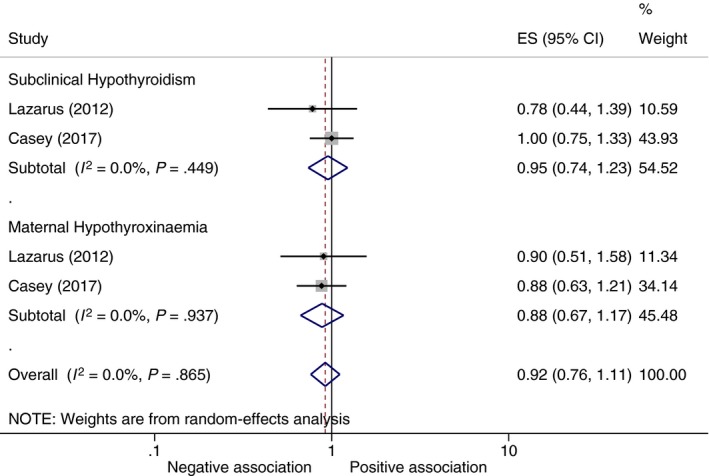
Meta‐analysis of RCTs on the effect of levothyroxine treatment for maternal subclinical hypothyroidism and hypothyroxinaemia on IQ of offspring. ES, Odds ratio point estimate; negative association, trait associated with decreased odds of neurodevelopmental impairment; positive association, trait associated with increased odds of neurodevelopmental impairment

We identified 2 RCTs that reported the effects of treatment of maternal thyroid hormone insufficiency with levothyroxine on the incidence of low IQ. Compared to children born to untreated mothers with subclinical hypothyroidism, there was no significant difference in IQ levels in children born to such mothers treated with levothyroxine (OR 0.95, 95% CI 0.74 to 1.23, *P* = .71) (Figure 4). Likewise, there was also no significant difference in IQ levels in children born to mothers with hypothyroxinaemia treated with levothyroxine and those who were not treated (OR 0.88, 95% CI 0.67 to 1.17, *P* = .38) (Figure 4).

## 4 DISCUSSION

This systematic review and meta‐analysis show an association between maternal subclinical hypothyroidism and hypothyroxinaemia during pregnancy and indicators of intellectual disability. There was no association between maternal thyroid hormone insufficiency and the risk of ADHD in offspring, and the effect of maternal thyroid hormone insufficiency on the risk of autism in the offspring was unclear. We also found no evidence that levothyroxine treatment of mothers with subclinical hypothyroidism or hypothyroxinaemia during pregnancy reduces the incidence of low IQ in children, although the number of studies with data able to contribute to this was small (n = 2).

During the course of our study, 2 systematic reviews with meta‐analyses on the association between maternal thyroid hormone insufficiency and neurodevelopmental disorders in offspring were published.^52^, ^53^ Compared to these previous systematic reviews, our study had a wider scope as we analysed multiple neurodevelopmental disorders (including autism and ADHD) in addition to indicators of intellectual disability, and performed meta‐analysis on RCTs of levothyroxine treatment for pregnant women with mild thyroid hormone insufficiency to improve neurological outcomes in offspring. We searched 9 mainstream databases (as well as 3 grey literature bases), as compared to 2 databases searched by Wang et al^53^ and 3 databases search by Fan et al,^52^ thus finding more eligible studies and increasing confidence that we have identified all available relevant evidence. Our finding of the association between maternal subclinical hypothyroidism and hypothyroxinaemia with indicators of intellectual disability in offspring is consistent with the results of the previous meta‐analyses, although our result did show a smaller magnitude of an effect.^52^, ^53^ Our meta‐analysis included more studies per meta‐analysis than the previous systematic reviews,^52^, ^53^ for example, Fan and colleagues^52^ included only 2 studies in their subclinical hypothyroidism meta‐analysis compared to the 11 studies included in our meta‐analysis. Wang et al^53^ did not assess the association between subclinical hypothyroidism and indicators of intellectual disability. In a previous systematic review, Chan and Boelaert^54^ analysed the effects of subclinical hypothyroidism and hypothyroxinaemia on obstetric outcomes; however, the study did not include meta‐analysis of child neurodevelopment outcomes.

The association between mild maternal thyroid hormone insufficiency (subclinical hypothyroidism and hypothyroxinaemia) and neurodevelopment in offspring found in this systematic review is biologically plausible. Thyroid hormones have an important impact at various stages of foetal neurological development, including proliferation and differentiation of neuronal precursors, neuronal migration and myelination.^55^ FT3, the active form of thyroxine, plays a key role in the process of neuronal migration in the brain by stimulating brain cells (particularly Cajal‐Retzius cells in the cerebellum) to produce Reelin, which in turn acts as a scaffold for migrating neurons.^55^ This is a major part in the formation of the cerebellum,^56^ the corpus callosum^57^ and the cerebral cortex,^55^ areas in the brain that are important in motor^58^, ^59^ and cognitive function.^60^ Thyroid hormone is also important in the process of white matter formation, stimulating the secretion of proteins relevant to myelination.^61^ Thus, mild maternal thyroid hormone insufficiency could influence neurodevelopment via these mechanisms. Indeed, recent imaging studies have demonstrated structural changes in different parts of the brain in children of women with thyroid hormone insufficiency in pregnancy.^44^, ^62^, ^63^, ^64^ Interestingly, the effect of thyroxine on neuronal migration is largely influenced by the α‐thyroid hormone receptor, which is expressed throughout foetal development, whilst myelination is largely influenced by the β‐thyroid hormone receptor which is expressed much later in development.^65^ Even though maternal thyroid hormone insufficiency in early gestation (before the foetal thyroid gland becomes functional) is potentially likely to have more deleterious effect on foetal neurodevelopment, our sensitivity analyses of the studies in which thyroid function was measured before 12 weeks failed to show an association between maternal subclinical hypothyroidism or hypothyroxinaemia and indicators of intellectual disability. The explanation for this observation is unclear but may be due to smaller sample sizes.

This systematic review found no association between maternal thyroid hormone insufficiency and offspring risk of ADHD while its impact on the offspring risk of autism was unclear. As some researchers have suggested autism appears to be linked to a lack of neuronal migration,^66^ and some studies suggest ADHD is linked to a lack of functional connectivity^67^ (hinting at a lack of white matter formation), it seems possible that hypothyroxinaemia in early pregnancy could lead to autism whilst hypothyroxinaemia in later pregnancy could lead to ADHD. As most of the studies measured fT4 between the first and second trimesters, this could explain the lack of an association found between maternal thyroid hormone insufficiency and ADHD. Future studies should explore the effects of maternal thyroid hormone insufficiency at different stages of pregnancy.

We were unable to demonstrate evidence of a beneficial effect of levothyroxine treatment in pregnant women with mild thyroid hormone insufficiency on neurodevelopmental outcomes in offspring. Considering that at an observational level both maternal hypothyroxinaemia and subclinical hypothyroidism were associated with indicators of intellectual disability, this result is surprising. It could be that the intervention was delivered too late in gestation (average gestational age at recruitment: Lazarus et al^7^ ~12 weeks, Casey et al^49^ ~17 weeks) for it to have a meaningful effect; however, both studies performed sensitivity analysis on when in pregnancy the intervention was delivered and found no different result. It is also possible that the studies measured the IQ of the children at too young an age (Lazarus et al^7^ at 3 years, Casey et al^49^ at 5 years), considering that the original Haddow et al^2^ study measured IQ at 8 years. Furthermore, there is a possibility that the association between hypothyroxinaemia and indicators of intellectual disability seen in the observational studies is mediated by iodine deficiency,^68^ and thus not completely ameliorated by levothyroxine. Finally, it is also possible that the RCTs were not big enough to see an effect. Therefore, further large RCTs with intervention earlier in pregnancy and longer follow‐up of children are needed to resolve this issue.

The current guideline from the American Thyroid Association recommends levothyroxine treatment for pregnant women with subclinical hypothyroidism in the presence of thyroid peroxidase antibodies or serum TSH greater than 10 mIU/L but not for maternal hypothyroxinaemia.^69^ In contrast, the guideline from the European Thyroid Association recommends treating maternal subclinical hypothyroidism as well as maternal hypothyroxinaemia, the latter if diagnosed during the first trimester.^70^ Neither Association has recommended universal screening for subclinical hypothyroidism or maternal hypothyroxinaemia during pregnancy, owing to a lack of robust evidence for the benefit from screening and treating mild thyroid hormone insufficiency.^69^, ^70^ The findings of this systematic review support the Associations' recommendations and support the guideline from the American Thyroid Association on not treating isolated maternal hypothyroxinaemia. This systematic review does not support the Associations' recommendations^69^, ^70^ of treating maternal subclinical hypothyroidism in pregnancy to prevent low IQ; however, clinicians may wish to treat subclinical hypothyroidism in pregnancy to prevent other adverse obstetric outcomes like miscarriage and premature birth.^71^

This study has several limitations. Firstly, there was heterogeneity between the studies, in terms of study population (together with differential iodine status of the populations), gestational age at the time of thyroid dysfunction, different definitions of thyroid dysfunction, whether or not patients on thyroxine included in the cohort, age of offspring at the time of neuropsychological assessment and measures of neurodevelopmental disorders (Table S1). There are also the inherent problems linked to observational studies, which formed the majority of the studies analysed, such as selection bias. Secondly, it was difficult to categorize the type of thyroid hormone insufficiency for some studies. For example, studies reported by Haddow et al^2^ and Päkkilä et al^48^ classified thyroid dysfunction based on TSH levels rather than the combination of TSH and fT4 levels. Therefore, although we included these as subclinical hypothyroidism studies, it is possible that a small number of subjects with high TSH levels had overt hypothyroidism. However, sensitivity analyses in which we removed these studies did not change the overall results of the meta‐analysis. Likewise, in the RCT reported by Lazarus et al,^7^ 23 of the mothers in the hypothyroxinaemia group also had both elevated TSH levels in addition to low fT4 levels, thus constituting overt hypothyroidism, though they were in the minority and unlikely to have impacted on the overall results. Thirdly, 5 observational studies also explicitly included participants being treated with levothyroxine for hypothyroidism within the case group,^2^, ^5^, ^23^, ^37^, ^50^ making the direct comparison with other studies where the treatment of participants is unclear difficult. Also, different studies had different exclusion criteria, for example 2 studies only included participants with known thyroid disease,^5^, ^20^ whilst others explicitly excluded participants with known thyroid disease.^28^, ^32^ Finally, several studies report thyroid function as continuous outcomes rather than dichotomous. Where possible, we converted results from such studies into odds ratios using 2 different methods,^14^, ^15^, ^16^, ^17^ and there was no difference in interpretation between the 2 results generated. However, we excluded 5 studies from meta‐analysis as it was not possible to convert their results to odds ratios.

In conclusion, our systematic review and meta‐analysis showed an association between mild maternal thyroid hormone insufficiency (subclinical hypothyroidism and hypothyroxinaemia) in pregnancy and impaired neuropsychological development in offspring. We found no evidence that levothyroxine treatment for maternal mild thyroid hormone insufficiency, including maternal hypothyroxinaemia and subclinical hypothyroidism, improves neurodevelopmental outcomes in children.

## CONFLICT OF INTEREST

The authors have nothing to disclose.
