# Supplementary material for: Maternal thyroid hormone insufficiency during pregnancy and risk of neurodevelopmental disorders in offspring: A systematic review and meta‐analysis
Source: Clin Endocrinol (Oxf). 2018 Feb 8;88(4):575–84. doi: 10.1111/cen.13550 (PMC5888183; doi:10.1111/cen.13550)
Supplement: Supplementary file 1 [file CEN-88-575-s001.docx]

**Supplementary section**

**Maternal thyroid hormone insufficiency in pregnancy and risk of neurodevelopmental disorders in offspring: A systematic review and meta-analysis**

William Thompson, Ginny Russell, Genevieve Baragwanath, Justin Matthews, Bijay Vaidya, Jo Thompson-Coon

Appendix 1: Search strategy for MEDLINE

Database: Ovid MEDLINE(R) In-Process & Other Non-Indexed Citations and Ovid MEDLINE(R) <1946 to Present>, Ovid MEDLINE(R) Daily Update <November 18, 2015>

Search Strategy:

--------------------------------------------------------------------------------

1 hypothyroid*.ti,ab. (28893)

2 hypothyroxin?emia.ti,ab. (456)

3 Thyroid Disease*.ti,ab. (11143)

4 Thyroid Hormone*.ti,ab. (30862)

5 Triiodothyronin*.ti,ab. (15160)

6 Thyroxin*.ti,ab. (26649)

7 Thyroid.ti,ab. (146841)

8 thyroid deficien*.ti,ab. (309)

9 Thyrotropin.ti,ab. (15626)

10 thyroid dysfunction.ti,ab. (3672)

11 Hashimoto*.ti,ab. (5822)

12 Thyroiditis.ti,ab. (11843)

13 1 or 2 or 3 or 4 or 5 or 6 or 7 or 8 or 9 or 10 or 11 or 12 (185372)

14 Autis*.ti,ab. (29378)

15 Language development disorder*.ti,ab. (36)

16 Child development disorder*.ti,ab. (7)

17 Attention Deficit Disorder with Hyperactivity.ti,ab. (233)

18 Asperger*.ti,ab. (1826)

19 Pervasive Development* Disorder*.ti,ab. (1843)

20 Social responsiveness scale.ti,ab. (214)

21 Social behavio?r.ti,ab. (7152)

22 childhood disintegrative disorder*.ti,ab. (70)

23 ASD.ti,ab. (11422)

24 ASC.ti,ab. (5335)

25 AD.ti,ab. (99735)

26 PDD.ti,ab. (2588)

27 ADHD.ti,ab. (16749)

28 Attention Deficit Hyperactivity Disorder.ti,ab. (17139)

29 Attention Deficit Disorder.ti,ab. (1543)

30 Hyperactivity Disorder.ti,ab. (17415)

31 Hyperkinetic disorder.ti,ab. (193)

32 Child Behavio?r disorder.ti,ab. (5)

33 Child Behavio?r disorders.ti,ab. (34)

34 Hyperkinesis.ti,ab. (678)

35 Neurodevelopment*.ti,ab. (18701)

36 Prenatal Exposure Delayed Effects/ or Brain/ or Nervous System/ (450441)

37 prenatal exposure delayed effects.ti,ab. (2)

38 Brain.ti,ab. (761560)

39 Nervous System.ti,ab. (215524)

40 neuropsychological.ti,ab. (36232)

41 neurocognitive.ti,ab. (12078)

42 neurobehavio?ral.ti,ab. (9323)

43 Cognition/ (72569)

44 cognition.ti,ab. (41565)

45 Developmental Disabilities/ (16439)

46 Developmental disabilities.ti,ab. (3169)

47 IQ.ti,ab. (16510)

48 intelligence quotient.ti,ab. (2166)

49 Intellectual Disability/ or Intelligence/ (67891)

50 intelligence.ti,ab. (24040)

51 intellectual disability.ti,ab. (6741)

52 mental* retard*.ti,ab. (30942)

53 mental* deficien*.ti,ab. (1775)

54 developmental disorder*.ti,ab. (6391)

55 learning disorder*.ti,ab. (922)

56 special education* need*.ti,ab. (235)

57 Global developmental delay.ti,ab. (533)

58 language disorder.ti,ab. (751)

59 language disorders.ti,ab. (1322)

60 Speech Sound Disorder.ti,ab. (86)

61 Stutter*.ti,ab. (3778)

62 Auditory Perception/ or Phonetics/ or Speech/ (52187)

63 Auditory Perception.ti,ab. (1173)

64 Phonetics.ti,ab. (449)

65 Speech.ti,ab. (60348)

66 Fluency Disorder*.ti,ab. (66)

67 communication disorder*.ti,ab. (1041)

68 dyslexia.ti,ab. (4206)

69 dyscalculia.ti,ab. (315)

70 Mathematics/ or Cognition Disorders/ (131979)

71 Mathematics.ti,ab. (4906)

72 cognition disorder*.ti,ab. (158)

73 cognition.ti,ab. (41565)

74 Reading Disorder*.ti,ab. (383)

75 spelling disorder*.ti,ab. (45)

76 Writing/ or Agraphia/ or Verbal Learning/ or Pattern Recognition, Visual/ or Reading/ (68671)

77 Writing.ti,ab. (19457)

78 Agraphia.ti,ab. (663)

79 Verbal Learning.ti,ab. (3580)

80 Pattern Recognition.ti,ab. (11614)

81 Reading.ti,ab. (92607)

82 Disorder of Arithmetic Skill*.ti,ab. (5)

83 Brain Diseases/ (50429)

84 brain disease*.ti,ab. (4303)

85 Disorder of scholastic skill*.ti,ab. (3)

86 Developmental Coordination Disorder.ti,ab. (694)

87 Motor Skills Disorders/ (2345)

88 Motor Skills Disorder*.ti,ab. (6)

89 Dyspraxia.ti,ab. (418)

90 Apraxias/ (2447)

91 Apraxia*.ti,ab. (3167)

92 Disorder of Motor function.ti,ab. (10)

93 Movement Disorders/ (14472)

94 movement disorder*.ti,ab. (10556)

95 Stereotyp* movement disorder*.ti,ab. (51)

96 Stereotyped Behavior/ (8135)

97 Stereotyp* behavio?r.ti,ab. (1993)

98 Tic disorder.ti,ab. (486)

99 tic disorders.ti,ab. (645)

100 Tourettes syndrome.ti,ab. (1571)

101 Tourette syndrome.ti,ab. (2363)

102 14 or 15 or 16 or 17 or 18 or 19 or 20 or 21 or 22 or 23 or 24 or 25 or 26 or 27 or 28 or 29 or 30 or 31 or 32 or 33 or 34 or 35 or 37 or 38 or 39 or 40 or 41 or 42 or 44 or 45 or 46 or 47 or 48 or 50 or 51 or 52 or 53 or 54 or 55 or 56 or 57 or 58 or 59 or 60 or 61 or 63 or 64 or 65 or 66 or 67 or 68 or 69 or 71 or 72 or 73 or 74 or 75 or 77 or 78 or 79 or 80 or 81 or 82 or 84 or 85 or 86 or 88 or 89 or 91 or 92 or 94 or 95 or 97 or 98 or 99 or 100 or 101 (1370075)

103 maternal.ti,ab. (190120)

104 mother*.ti,ab. (169278)

105 pregnan*.ti,ab. (404068)

106 gestation*.ti,ab. (162703)

107 uter*.ti,ab. (160526)

108 Placent*.ti,ab. (85965)

109 103 or 104 or 105 or 106 or 107 or 108 (807033)

110 13 and 102 and 109 (1663)

**Supplementary Figure 1:** Meta-analysis of studies on association between maternal subclinical hypothyroidism and indicators of intellectual disability in offspring (after excluding studies with mixed overt and subclinical hypothyroidism). ES=Odds ratio point estimate, negative association=trait associated with decreased odds of neurodevelopmental impairment, positive association=trait associated with increased odds of neurodevelopmental impairment.

**
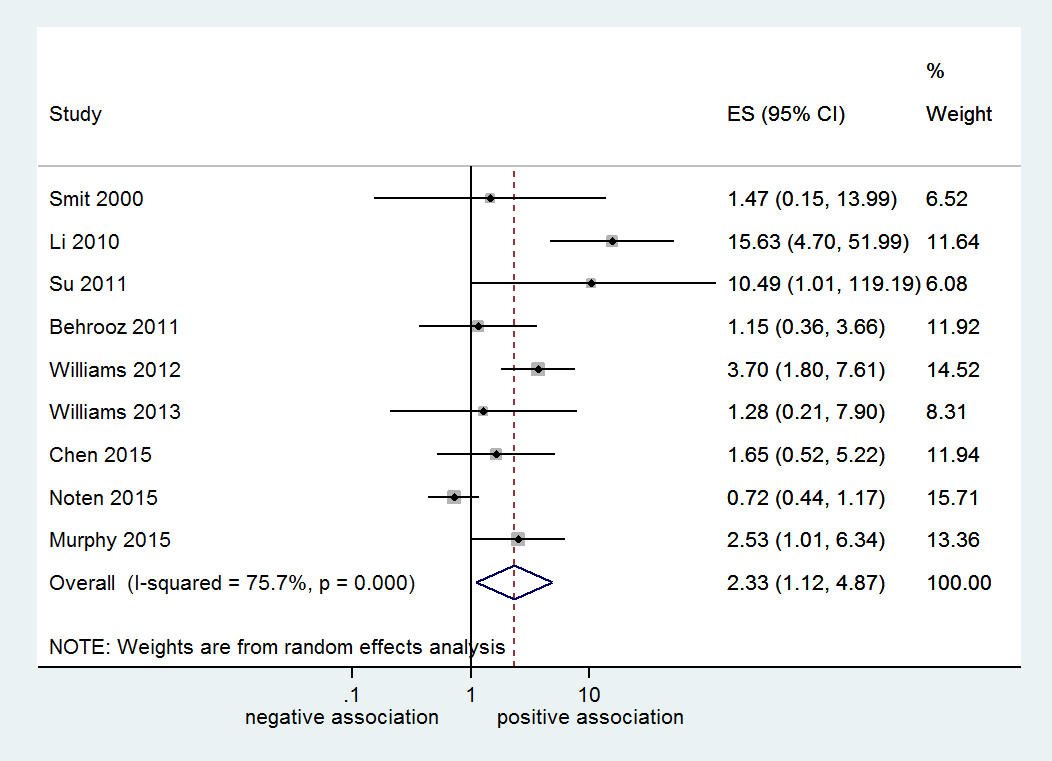
**

**Supplementary Figure 2:** Meta-analysis of studies on association between maternal overt hypothyroidism (based on hospital diagnosis) and autism in offspring. ES=Odds ratio point estimate, negative association=trait associated with decreased odds of neurodevelopmental impairment, positive association=trait associated with increased odds of neurodevelopmental impairment.

**
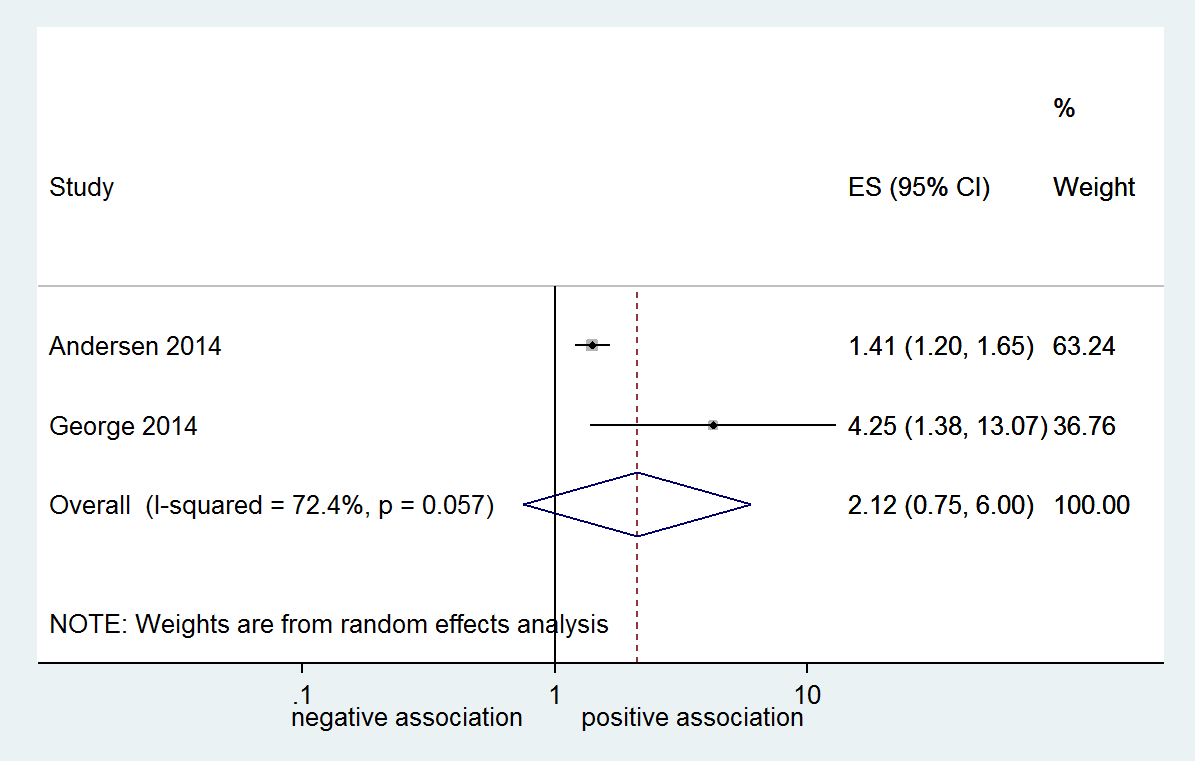
**

**Supplementary Figure 3:** Meta-analysis of studies on association between maternal subclinical hypothyroidism and ADHD in offspring. ES=Odds ratio point estimate, negative association=trait associated with decreased odds of neurodevelopmental impairment, positive association=trait associated with increased odds of neurodevelopmental impairment. **
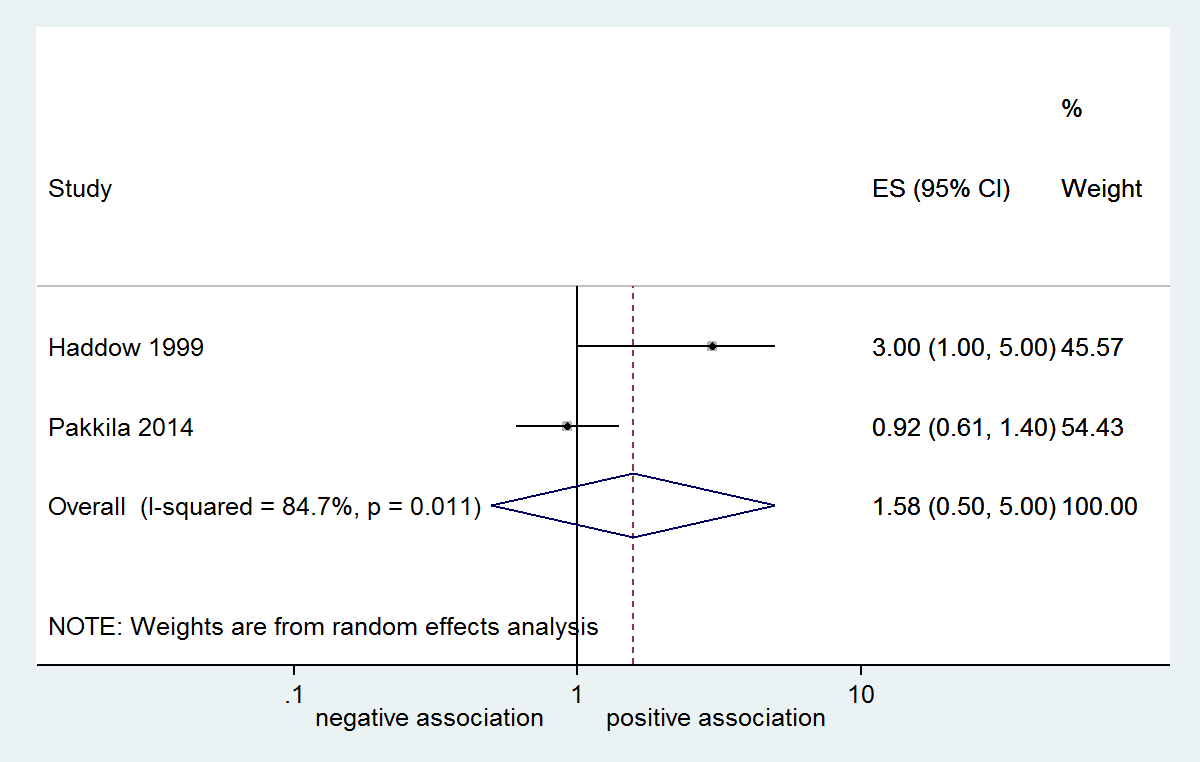
**

**Supplementary Figure 4:** Meta-analysis of studies on association between maternal hypothyroxinaemia and ADHD in offspring. ES=Odds ratio point estimate, negative association=trait associated with decreased odds of neurodevelopmental impairment, positive association=trait associated with increased odds of neurodevelopmental impairment.

**
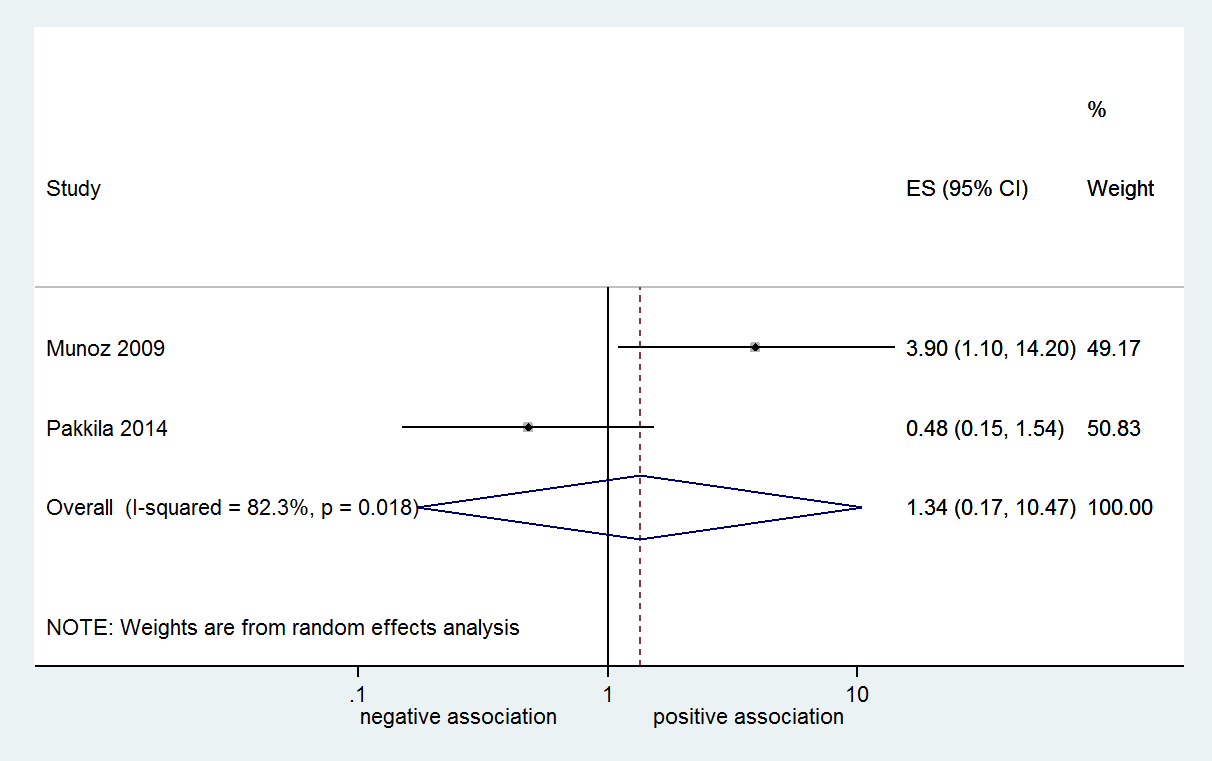
**

**Supplementary Figure 5:** Meta-analysis of studies on association between maternal subclinical hypothyroidism and indicators of intellectual disability in offspring (with results split between TSH measured in mothers before 12 weeks gestation (<12 weeks) and at or after 12 weeks gestation (=>12 weeks). As the point at which TSH was measured in Chen et al. 2015^26^ is not known, the study was not included in the meta-analysis. ES=Odds ratio point estimate, negative association=trait associated with decreased odds of neurodevelopmental impairment, positive association=trait associated with increased odds of neurodevelopmental impairment.
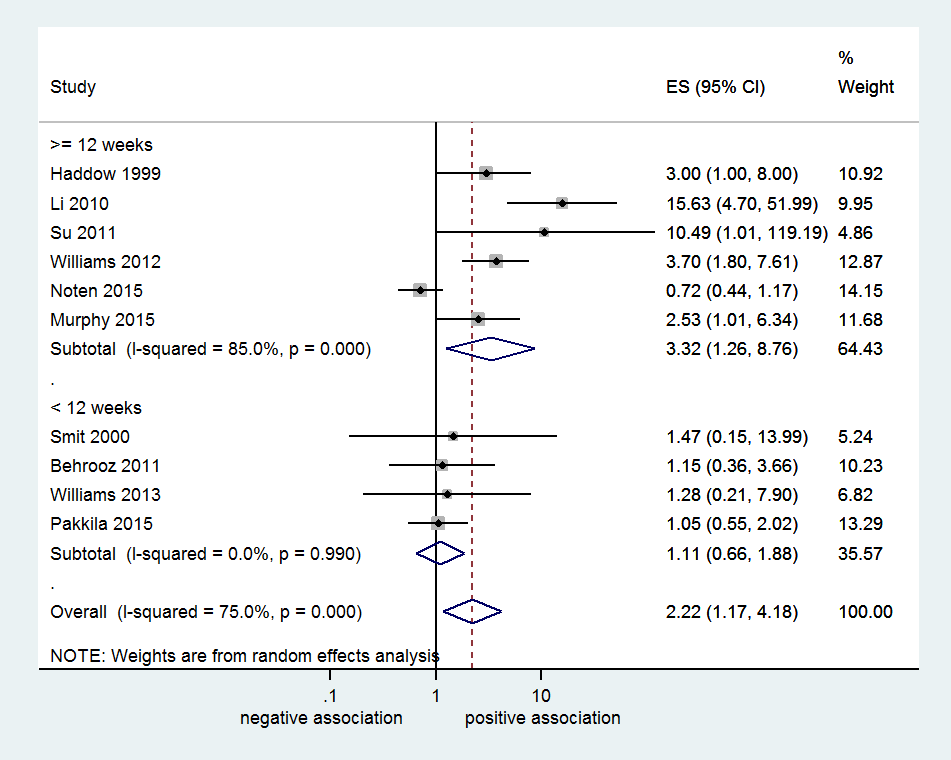


## Supplementary Figure 6: Meta-analysis of studies on association between maternal hypothyroxinaemia and indicators of intellectual disability in offspring (with results split between fT_4_ measured in mother before 12 weeks gestation (<12 weeks) and at or after 12 weeks gestation (=>12 weeks). ES=Odds ratio point estimate, negative association=trait associated with decreased odds of neurodevelopmental impairment, positive association=trait associated with increased odds of neurodevelopmental impairment.


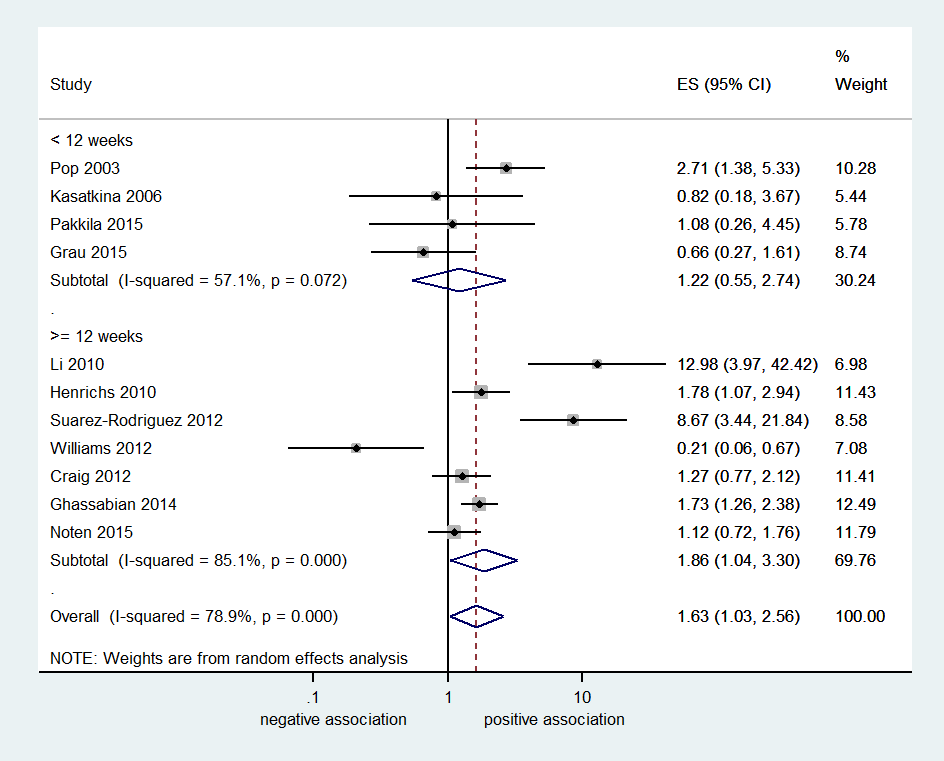


**Supplementary Table 1:** Characteristics of all studies included in the systematic review (sibling papers merged)

**Abbreviations:** ADHD=Attention deficit & hyperactivity disorder, Co=Continuous, DSM-IV=Diagnostic and statistical manual of mental disorders - fourth edition, HR=Hypothyroxinaemia, ICD=International classification of diseases, IQR=Interquartile range, NA=Not applicable, NK=Not known, OH=Overt hypothyroidism, RCT=Randomised controlled trial, SH=Subclinical hypothyroidism, Ref range=Reference range, TFT=Thyroid function tests, TSH=Thyroid stimulating hormone, tT4= Total thyroxine, WISC=Wechsler intelligence scale for children, WPPSI= Wechsler preschool and primary scale of intelligence

| **Author, year** | **Study type** | **Total number of participants tested for outcome** | **Country** | **Maternal thyroid dysfunction** | **Gestation at TFT** | **Criteria for thyroid dysfunction** | **Child age at assessment** | **Neurodevelopment outcome measures** |
| --- | --- | --- | --- | --- | --- | --- | --- | --- |
| Pop, 1999^19^ | Cohort | 220 | Netherlands | HR | 12 and 32 weeks | 10th percentile fT4 (<10.4 pmol/l) and 5th percentile fT4 (<9.8 pmol/l) | 10 months | Bayley Scales of Infant Development |
| Haddow, 1999^2^  /Klein, 2001^21^ | Matched Cohort | 186 | USA | SH/OH | 17 weeks | 99.7^th^ percentile TSH or 98th percentile TSH with tT4 <99.7nmol/l | 7-9 years | WISC, 3rd edition and Conners Continuous Performance test |
| Smit, 2000^20^ | Cohort | 20 | Netherlands | SH | 1^st^, 2^nd^ and 3^rd^ trimesters | TSH>2-2.70 mU/l depending on trimester | 6, 12 and 24 months | Bayley Scale of Infant Development (Touwens method at 6 and 12 months, Hempel's method at 24 months) |
| Pop, 2003^22^ | Matched Cohort | 125 | Netherlands | HR | 12, 24 and 32 weeks | fT4 <10th Percentile (12.10 pmol/l) | 1-2 years | Bayley Scale of Infant Development |
| Kasatkina, 2006^23^ | Cohort | 35 | Russia | HR | 1^st^ and 3^rd^ trimesters | fT4 <12.0 pmol/l | 6,9 and 12 months | Gnome method, in particular the Coefficient of Mental Development |
| Kooistra, 2006^24^ | Matched Cohort | 204 | Netherlands | HR | 12, 24 and 32 weeks | fT4 <10th Percentile (11.40-12.0 pmol/l) | 3 weeks | Neonatal Behavioural Assessment Scale |
| Oken, 2009^26^ | Cohort | 500 | USA | SH and Co tT4 | 10.2 weeks | SH = TSH >2.50 mU/l, Co tT4 = tT4 ref range 57.92-140.28 nmol/l | 6 months and 3 years | Visual Recognition Memory at 6 months, Peabody Picture Vocabulary Test and Wide Range Assessment of Visual Motor Ability at 3 years |
| Munoz, 2009^25^ | Matched Cohort | 59 | Spain | HR | 3^rd^ trimester | fT4 <10th Percentile (10.17 pmol/l) | 2 years | Parental report based on the criteria in DSM-IV for ADHD |
| Henrichs, 2010^27^ | Cohort | 3659 | Netherlands | HR and  Co TSH | 13.3 weeks | HR=fT4 10th percentile (<11.76 pmol/l) and 5th percentile (<10.96 pmol/l),  Co TSH= TSH ref range 0.03-2.50 mU/l | 18 and 30 months | MacArthur Communicative Development Inventory at 18 months, Language Development Survey at 30 months |
| Li, 2010^28^ | Matched Cohort | 213 | China | SH and  HR | 16 to 20 weeks | SH=TSH >97.50th percentile (4.21 mU/l), HR= tT4<2.50th percentile (101.79 nmol/l) | 25-30 months | Bayley Scale of Infant Development |
| Ghassabian, 2011^30^ | Cohort | 3736 | Netherlands | Co fT4  and TSH | < 18 weeks | Co fT4=<10th percentile (11.76 pmol/l)  Co TSH= range 0.03-2.50 mU/l | 18 months and 3 years | The Child Behaviour Checklist was used by mothers at 18 months and by both parents at 3 years |
| Su, 2011^31^ | Cohort | 1017 | China | OH, SH and HR | < 20 weeks | fT4=<5th percentile (10.40-8.51 pmol/l) gestation dependent,  TSH=>95th percentile (3.77-4.35 mU/l) gestation dependent | 6 months | Chinese Bayley scales for Infant development |
| Chevrier, 2011^29^ | Cohort | 287 | USA | Co fT4  and TSH | 27 weeks | fT4= Low fT4 defined as <6.44 pmol/l  TSH= High TSH >2.50 mU/l | 6 months and 1, 2 and 5 years | Bayley Scales of Infant Development at 6, 12 and 24 months, WPPSI 3rd edition and Conner’s Kiddie Continuous Performance Test at 5 years |
| Behrooz, 2011^5^ | Cohort | 38 | Iran | SH | 10 weeks | SH= TSH >3.0 mU/l | 4-14.5 years | WISC |
| Williams, 2012^33^ | Cohort | 166 | UK | SH and HR | ±1 hour after birth | SH=TSH >3.0 mU/l,  HR=fT4 ≤10th percentile (11.6 pmol/l) or tT4 ≤10th percentile (108.4 nmol/) | 5.5 years | The McCarthy Scales of Children’s Abilities |
| Suárez-Rodríguez, 2012^32^ | Cohort | 70 | Spain | HR | 37 weeks | fT4 <10th percentile (9.5 pmol/l) | 38 months and 5 years | The McCarthy Scales of Children’s Abilities |
| Craig, 2012^6^ | Matched Cohort | 196 | USA | HR | 2^nd^ trimester | fT4 <3rd Percentile (11.84 pmol/l) | 2 years | Bayley’s Scale of Infant Development III |
| Lazarus, 2012^7^ | RCT | 794 | UK and Italy | SH and  HR | <16 weeks (median 12.3 weeks) | TSH >97.5th percentile, fT4 <2.5th percentile | 3 years | WPPSI, third edition |
| Williams, 2013^36^ | Cohort | 97 | UK | SH | 10 weeks, 34 weeks and at delivery | TSH ≥2.50 mU/l (at 10 weeks) or ≥2.50 mU/l (at 34 weeks) | 5.5 years | The McCarthy Scales of Children’s Abilities |
| Julvez, 2013^35^ | Cohort | 1761 | Spain | Co fT4  and TSH | 13 weeks | Co fT4=10th percentile (fT4 <8.89 pmol/l), 5th percentile (fT4 <8.39 pmol/l) or 2.5th percentile (fT4 <8.10 pmol/l)  Co TSH= 90th percentile (TSH >2.59 mU/l), 95th percentile (TSH >3.20 mU/l) or 97.5th percentile (TSH >4.18 mU/l) | 11-23 months | Bayley Scales of Infant Development |
| Finken, 2013^34^ | Cohort | 1765 | Netherlands | Co fT4  and TSH | Median 90 days (IQR 83-100) | Co fT4= fT4 10th percentile (<7.70 pmol/l), 5th percentile (<7.0 pmol/l) or 2.5th percentile (<6.50 pmol/l)  Co TSH=90th percentile (>2.30 mU/l), 95th percentile (>2.90 mU/l) or 97.5th percentile (>3.80 mU/l) | 5-6 years | The Amsterdam Neuropsychological Tasks (ANT), a series of video game style tests measuring visuo-motor skills, such as baseline speed (the first task) |
| Roman, 2013^3^ | Cohort | 4039 | Netherlands | HR | Mean 13.4 weeks (range 5.9-17.9) | fT4 <10th percentile (11.82 pmol/l) or <5th percentile (10.99 pmol/l) | 6 years | Pervasive Developmental Problems on the Child Behavior Checklist for Toddlers (CBCL 1.5-5), parent reported |
| Andersen, 2013^37^ | Cohort | 857014 | Denmark | OH | NA | Hospital Diagnosis (ICD-8 and ICD-10) | 3 years | Hospital diagnosis and diagnosis in the Danish Psychiatric Central Registry of ADHD and autism. Also, prescription of ADHD medication. |
| Ghassabian, 2014^38^/ Korevaar, 2016^51^ | Cohort | 3737/5647 | Netherlands | HR and SH | 13.5/13.2 weeks | HR= fT4<5th percentile (10.99 pmol/l),  SH= TSH >2.50 mU/l | 6 years | Snijders-Oomen Niet-verbale intelligentie test, revisie (Mosaics and Categories) |
| Yau, 2014^39^ | Nested Case-Control | 272 | USA | Co TSH | 15 to 19 weeks | Neurodevelopment measured first, then compared to TSH levels (TSH in general population 0.42-3.69 mU/l) | 3-4 years | Autism diagnosis based on DSM-IV |
| George, 2014^50^ | Case-Control | 343 | India | OH | NA | Hospital Diagnosis | 2-6 years | Childhood Autism Rating Scale |
| Pakkila, 2014^18^ | Cohort | 5131 | Finland | SH and HR | Mean 10.7 weeks | HR=fT4 <11.40-11.10 pmol/l gestation dependent,  SH= TSH >3.10-3.50 mU/l gestation dependent | 8 years | The Rutters B2 scale |
| Korevaar, 2015^44^ | Cohort | 3839 | Netherlands | Co fT4  and TSH | Median 13.2 weeks (range 9.8-17.5) | fT4 ref range 10.4-22 pmol/l,  TSH ref range 0.03-4.04 mU/l | 6 years | Snijders-Oomen Niet-verbale intelligentie test, revisie (Mosaics and Categories) |
| Modesto, 2015^45^ | Cohort | 3873 | Netherlands | HR | Mean 13.6 weeks | fT4 <5th percentile (10.94 pmol/l) | 8 years | The Conners’ Parent Rating Scale-Revised Short Form (mother ranked) |
| Grau, 2015^43^ | Cohort | 455 | Spain | HR | 1^st^ and 2^nd^ trimesters | <10th Percentile (13.7-11.5 pmol/l depending on trimester) | 1 and 6-8 years | Brunet-Lezine scale and WISC-IV |
| Ganaie, 2015^42^ | Cohort | 133 | India | OH | 1^st^, 2^nd^ and 3^rd^ trimesters | Hospital Diagnosis | 3-4 weeks | Neonatal Behavioral Assessment Scale |
| Pakkila, 2015^48^ | Cohort | 5295 | Finland | HR, SH and OH | Mean 10.7 weeks | HR=fT4<11.4-11.09 pmol/l depending upon trimester,  SH=TSH>3.10-3.50 mU/l depending upon trimester | 8 and 16 years | Strengths and Weaknesses of ADHD Symptoms and Normal Behavior, Teacher reported child school performance, Youth Self Report and WISC-Revised |
| Murphy, 2015^46^ | Cohort | 70 | Ireland | SH | 14 and 36 weeks | TSH >98th percentile (>4.0-3.50 mU/l) | 7-8 years | WISC-IV |
| Chen, 2015^41^ | Cohort | 212 | China | SH | NK | TSH>3.47-4.99 mIU/l | 12-24 months | The Gesell development test (as revised by the Beijing Child’s Health Care Unit) |
| Noten, 2015^47^ | Cohort | 1196 | Netherlands | HR and SH | Median 12.9 weeks | HR=fT4<10^th^ percentile (8.06 pmol/l)  SH=TSH>90^th^ percentile (2.03 mU/l) | 5 years | The Central Institute for Test Development (CITO) language and arithmetic tests |
| Brown, 2015^40^ | Cohort | 967 | Finland | HR, SH and OH | 1^st^ and 2^nd^ trimesters | HR= fT4 <5^th^ percentile (12.61 pmol/l),  SH=TSH>5^th^ percentile (3.1 mU/l), OH=TSH >3.1 mU/l and fT4<12.61pmol/l | 0-18 years | Childhood Autism (ICD-10) diagnosis |
| Casey, 2017^49^ | RCT | 1203 | USA | SH and HR | <20 weeks  (Mean 16.7 weeks for SH and 17.8 weeks for HR) | HR=fT4<11 pmol/l  SH=> TSH 97.5^th^ percentile (3.00 mU/l) | 5 years | WPPSI-III |

**Supplementary Table 2**: The Downs and Black Checklist^13^ for assessing study quality

| **Question number** | **Checklist question** |
| --- | --- |
| Q 1 | Is the hypothesis/aim/objective of the study clearly described? Must be explicit. |
| Q 2 | Are the main outcomes to be measured clearly described in the Introduction or Methods section? If the main outcomes are first mentioned in the Results section, the question should be answered no. |
| Q 3 | Are the characteristics of the patients included in the study clearly described? In cohort studies and trials, inclusion and/or exclusion criteria should be given. In case-control studies, a case-definition and the source for controls should be given. |
| Q 4 | Are the interventions of interest clearly described? Treatments and placebo (where relevant) that are to be compared should be clearly described. |
| Q 5 | Are the distributions of principal confounders in each group of subjects to be compared clearly described? A list of principal confounders is provided. |
| Q 6 | Are the main findings of the study clearly described? Simple outcome data (including denominators and numerators) should be reported for all major findings so that the reader can check the major analyses and conclusions. |
| Q 7 | Does the study provide estimates of the random variability in the data for the main outcomes? In non-normally distributed data the inter-quartile range of results should be reported. In normally distributed data the standard error, standard deviation or confidence intervals should be reported. |
| Q 8 | Have all important adverse events that may be a consequence of the intervention been reported? This should be answered yes if the study demonstrates that there was a comprehensive attempt to measure adverse events. |
| Q 9 | Have the characteristics of patients lost to follow-up been described? This should be answered yes where there were no losses to follow-up or where losses to follow-up were so small that findings would be unaffected by their inclusion. This should be answered no where a study does not report the number of patients lost to follow-up. |
| Q 10 | Have actual probability values been reported (e.g. 0.035 rather than <0.05) for the main outcomes except where the probability value is less than 0.001? |
| Q 11 | Were the subjects asked to participate in the study representative of the entire population from which they were recruited? The study must identify the source population for patients and describe how the patients were selected. |
| Q 12 | Were those subjects who were prepared to participate representative of the entire population from which they were recruited? The proportion of those asked who agreed should be stated. |
| Q 13 | Were the staff, places, and facilities where the patients were treated, representative of the treatment the majority of patients receive? For the question to be answered yes the study should demonstrate that the intervention was representative of that in use in the source population. Must state type of hospital and country for yes. |
| Q 14 | Was an attempt made to blind study subjects to the intervention they have received? For studies where the patients would have no way of knowing which intervention they received, this should be answered yes. |
| Q 15 | Was an attempt made to blind those measuring the main outcomes of the intervention? Must be explicit. |
| Q 16 | If any of the results of the study were based on “data dredging”, was this made clear? Any analyses that had not been planned at the outset of the study should be clearly indicated. |
| Q 17 | In trials and cohort studies, do the analyses adjust for different lengths of follow-up of patients, or in case-control studies, is the time period between the intervention and outcome the same for cases and controls? Where follow-up was the same for all study patients the answer should yes. Studies where differences in follow-up are ignored should be answered no. |
| Q 18 | Were the statistical tests used to assess the main outcomes appropriate? The statistical techniques used must be appropriate to the data. |
| Q 19 | Was compliance with the intervention/s reliable? Where there was noncompliance with the allocated treatment or where there was contamination of one group, the question should be answered no. |
| Q 20 | Were the main outcome measures used accurate (valid and reliable)? Where outcome measures are clearly described, which refer to other work or that demonstrates the outcome measures are accurate should be answered yes. |
| Q 21 | Were the patients in different intervention groups (trials and cohort studies) or were the cases and controls (case-control studies) recruited from the same population? For example, patients for all comparison groups should be selected from the same hospital. The question should be answered unable to determine for cohort and case control studies where there is no information. |
| Q 22 | Were study subjects in different intervention groups (trials and cohort studies) or were the cases and controls (case-control studies) recruited over the same time? For a study which does not specify the time period over which patients were recruited, the question should be answered as unable to determine. |
| Q 23 | Were study subjects randomised to intervention groups? Studies which state that subjects were randomised should be answered yes except where method of randomisation would not ensure random allocation. |
| Q 24 | Was the randomised intervention assignment concealed from both patients and health care staff until recruitment was complete and irrevocable? All non-randomised studies should be answered no. If assignment was concealed from patients but not from staff, it should be answered no. |
| Q 25 | Was there adequate adjustment for confounding in the analyses from which the main findings were drawn? In nonrandomised studies if the effect of the main confounders was not investigated or no adjustment was made in the final analyses the question should be answered as no. |
| Q 26 | Were losses of patients to follow-up taken into account? If the numbers of patients lost to follow-up are not reported, the question should be answered as unable to determine. |
| Q 27 | Did the study have sufficient power to detect a clinically important effect where the probability value for a difference being due to chance is <5%? Sample sizes have been calculated to detect a difference of x% and y%. |

**Supplementary Table 3**: Study Quality Criteria in observational studies included in the systematic review. The rows are questions on the Downs and Black Checklist (Supplementary Table 2), the Columns are the studies. 1 = good study design criteria met, 0 = good study design criteria not met, UTD = unable to determine.

| Study ID | Haddow | Pop 1999 | Smit | Klein | Pop  2003 | Kasatkina | Kooistra | Munoz | Oken | Henrichs | Li | Behrooz | Chevrier |
| --- | --- | --- | --- | --- | --- | --- | --- | --- | --- | --- | --- | --- | --- |
| Q 1 | 1 | 1 | 1 | 1 | 1 | 1 | 1 | 1 | 1 | 1 | 1 | 1 | 1 |
| Q 2 | 1 | 0 | 1 | 0 | 1 | 0 | 1 | 1 | 1 | 1 | 1 | 1 | 1 |
| Q 3 | 1 | 1 | 1 | 0 | 1 | 1 | 1 | 1 | 1 | 1 | 1 | 1 | 1 |
| Q 4 | 1 | 1 | 1 | 1 | 1 | 1 | 1 | 1 | 1 | 1 | 1 | 1 | 1 |
| Q 5 | 1 | 1 | 1 | 1 | 1 | 0 | 1 | 1 | 1 | 1 | 1 | 1 | 1 |
| Q 6 | 1 | 0 | 0 | 1 | 0 | 1 | 1 | 1 | 1 | 1 | 1 | 0 | 1 |
| Q 7 | 1 | 1 | 1 | 1 | 1 | 1 | 1 | 1 | 1 | 1 | 1 | 1 | 1 |
| Q 8 | 1 | 0 | 1 | 0 | 0 | 0 | 0 | 0 | 0 | 0 | 0 | 0 | 0 |
| Q 9 | 0 | 1 | 1 | 1 | 1 | UTD | 1 | UTD | 1 | 1 | 0 | 0 | 1 |
| Q 10 | 1 | 1 | 1 | 1 | 1 | 1 | UTD | 1 | 0 | 1 | 1 | 1 | 0 |
| Q 11 | 1 | 1 | 0 | 0 | 1 | UTD | 1 | 1 | 1 | 1 | 1 | 1 | 1 |
| Q 12 | 1 | 1 | 1 | 0 | 1 | UTD | 1 | 0 | 1 | 1 | 1 | 1 | 1 |
| Q 13 | 0 | 1 | 1 | 0 | 1 | 1 | 1 | 1 | 1 | 0 | 1 | 1 | 1 |
| Q 14 | 0 | 0 | 0 | 0 | 0 | 0 | 0 | 0 | 0 | 0 | 0 | 0 | 0 |
| Q 15 | 1 | 1 | 1 | 0 | 1 | 0 | 0 | 0 | 0 | 0 | 1 | 1 | 1 |
| Q 16 | 0 | 0 | 1 | 0 | 1 | 0 | 0 | 1 | 1 | 1 | 1 | 1 | 1 |
| Q 17 | 1 | 1 | 1 | 1 | 1 | 1 | 1 | 1 | 1 | 1 | 1 | 1 | 1 |
| Q 18 | 1 | 1 | 1 | 1 | 1 | 1 | 1 | 1 | 1 | 1 | 1 | 1 | 1 |
| Q 19 | 0 | 1 | 0 | 0 | 1 | 0 | 1 | 0 | 1 | 0 | 0 | 0 | 0 |
| Q 20 | 1 | 1 | 1 | 1 | 1 | 1 | 1 | 1 | 1 | 1 | 1 | 1 | 1 |
| Q 21 | 1 | 1 | UTD | UTD | 1 | UTD | 1 | UTD | 1 | 1 | 1 | 1 | 1 |
| Q 22 | 1 | 1 | 1 | UTD | 1 | 1 | 1 | 1 | 1 | 1 | 1 | 1 | 1 |
| Q 23 | 0 | 0 | 0 | 0 | 0 | 0 | 0 | 0 | 0 | 0 | 0 | 0 | 0 |
| Q 24 | 0 | 0 | 0 | 0 | 0 | 0 | 0 | 0 | 0 | 0 | 0 | 0 | 0 |
| Q 25 | 1 | 1 | 1 | 1 | 0 | 0 | 1 | 1 | 1 | 1 | 1 | 1 | 1 |
| Q 26 | UTD | 1 | 0 | 1 | 0 | 0 | 1 | 0 | 1 | 1 | 0 | 0 | 1 |
| Q 27 | 1 | UTD | UTD | UTD | UTD | 0 | UTD | UTD | UTD | UTD | UTD | UTD | UTD |
| Study ID | Ghassabian 2011 | Su | Craig | Suarez | Williams 2012 | Andersen | Finken | Julvez | Roman | Williams 2013 | George | Ghassabian 2014 | Pakkila 2014 |
| Q 1 | 1 | 1 | 1 | 1 | 1 | 1 | 1 | 1 | 1 | 1 | 1 | 1 | 1 |
| Q 2 | 1 | 1 | 1 | 1 | 1 | 1 | 1 | 1 | 1 | 1 | 1 | 1 | 1 |
| Q 3 | 1 | 1 | 1 | 1 | 1 | 1 | 1 | 1 | 1 | 1 | 1 | 1 | 1 |
| Q 4 | 1 | 1 | 1 | 0 | 1 | 1 | 1 | 0 | 1 | 1 | 1 | 1 | 1 |
| Q 5 | 1 | 1 | 1 | 1 | 1 | 1 | 1 | 1 | 1 | 1 | 0 | 1 | 1 |
| Q 6 | 1 | 1 | 1 | 1 | 1 | 1 | 1 | 1 | 1 | 1 | 1 | 1 | 1 |
| Q 7 | 1 | 1 | 1 | 1 | 1 | 1 | 1 | 1 | 1 | 1 | 1 | 1 | 1 |
| Q 8 | 0 | 1 | 0 | 0 | 0 | 0 | 0 | 0 | 0 | 1 | 1 | 0 | 0 |
| Q 9 | 1 | 1 | 1 | 1 | 0 | 1 | 1 | 1 | 1 | 1 | UTD | 1 | 1 |
| Q 10 | 1 | 1 | 1 | 1 | 1 | 0 | 1 | UTD | 1 | 1 | 1 | 1 | 0 |
| Q 11 | 1 | 1 | 1 | 1 | 1 | 1 | 1 | 1 | 1 | 1 | 1 | 1 | 1 |
| Q 12 | 1 | 1 | 1 | 1 | 1 | 1 | 1 | 1 | 1 | 1 | 1 | 1 | 1 |
| Q 13 | 1 | 1 | 1 | 1 | 1 | 1 | 1 | 1 | 1 | 1 | 1 | 1 | 1 |
| Q 14 | 0 | 0 | 0 | 0 | 0 | 0 | 0 | 0 | 0 | 0 | 0 | 0 | 0 |
| Q 15 | 0 | 1 | 1 | 0 | 0 | 1 | 0 | 1 | 0 | 0 | 0 | 0 | 0 |
| Q 16 | 1 | 1 | 1 | 0 | 1 | 1 | 1 | 1 | 1 | 1 | 1 | 1 | UTD |
| Q 17 | 1 | 1 | 1 | 1 | 1 | 1 | 1 | 1 | 1 | 1 | 1 | 1 | 1 |
| Q 18 | 1 | 1 | 1 | 1 | 1 | 1 | 1 | 1 | 1 | 1 | 1 | 1 | 1 |
| Q 19 | 1 | 0 | 0 | 0 | 0 | 0 | 1 | 0 | 0 | 0 | 0 | 1 | 1 |
| Q 20 | 1 | 1 | 1 | 1 | 1 | 1 | 1 | 1 | 1 | 1 | 1 | 1 | 1 |
| Q 21 | 1 | 1 | 1 | 1 | 1 | 1 | 1 | 1 | 1 | 1 | 0 | 1 | 1 |
| Q 22 | 1 | 1 | 1 | 1 | 1 | 1 | 1 | 1 | 1 | 1 | UTD | 1 | 1 |
| Q 23 | 0 | 0 | 0 | 0 | 0 | 0 | 0 | 0 | 0 | 0 | 0 | 0 | 0 |
| Q 24 | 0 | 0 | 0 | 0 | 0 | 0 | 0 | 0 | 0 | 0 | 0 | 0 | 0 |
| Q 25 | 1 | 1 | 1 | 1 | 1 | 1 | 1 | 1 | 1 | 1 | 1 | 1 | 1 |
| Q 26 | 1 | 1 | 1 | 0 | 1 | 0 | 0 | 1 | 1 | 0 | UTD | 0 | 1 |
| Q 27 | UTD | UTD | UTD | 0 | UTD | UTD | UTD | 1 | UTD | UTD | UTD | 1 | UTD |

| Study ID | Pakkila  2015 | Yau | Chen | Ganaie | Grau | Korevaar 2015 | Modesto | Murphy | Noten | Brown | Korevaar 2016 |
| --- | --- | --- | --- | --- | --- | --- | --- | --- | --- | --- | --- |
| Q 1 | 1 | 1 | 1 | 1 | 1 | 1 | 1 | 1 | 1 | 1 | 1 |
| Q 2 | 1 | 1 | 1 | 1 | 1 | 1 | 1 | 1 | 1 | 1 | 1 |
| Q 3 | 1 | 1 | 1 | 1 | 1 |  | 1 | 1 | 1 | 1 | 1 |
| Q 4 | 0 | 1 | 0 | 1 | 1 | 1 | 1 | 1 | 1 | 1 | 1 |
| Q 5 | 1 | 1 | 1 | 0 | 1 | 1 | 1 | 1 | 1 | 1 | 1 |
| Q 6 | 1 | 1 | 1 | 1 | 1 | 1 | 1 | 1 | 1 | 1 | 1 |
| Q 7 | 1 | 1 | 1 | 1 | 1 | 1 | 1 | 1 | 1 | 1 | 1 |
| Q 8 | 0 | 0 | 0 | 0 | 0 | 0 | 0 | 0 | 0 | 0 | 1 |
| Q 9 | 1 | 0 | 1 | UTD | 1 | 0 | 1 | 1 | 1 | 0 | 0 |
| Q 10 | 0 | 1 | 1 | 1 | 1 | 1 | 1 | 1 | 1 | 1 | 1 |
| Q 11 | 1 | 1 | 1 | UTD | 1 | 1 | 1 | 1 | 1 | 1 | 1 |
| Q 12 | 1 | 1 | 1 | UTD | 1 | 1 | 1 | 1 | 1 | 1 | 1 |
| Q 13 | 1 | 0 | 1 | 0 | 1 | 1 | 1 | 1 | 1 | 1 | 1 |
| Q 14 | 0 | 0 | 0 | 0 | 0 | 0 | 0 | 0 | 0 | 0 | 0 |
| Q 15 | 0 | 0 | 1 | 0 | 0 | 1 | 0 | 1 | 0 | 1 | 1 |
| Q 16 | 0 | 1 | 1 | 1 | 0 | 1 | 0 | 1 | 1 | 1 | 1 |
| Q 17 | 1 | 1 | 1 | 1 | 1 | 1 | 1 | 1 | 1 | 1 | 1 |
| Q 18 | 1 | 1 | 1 | 1 | 1 | 1 | 1 | 1 | 1 | 1 | 1 |
| Q 19 | 0 | 1 | 0 | 1 | 0 | 0 | 0 | 0 | 0 | 0 | 0 |
| Q 20 | 1 | 1 | 1 | 1 | 1 | 1 | 1 | 1 | 1 | 1 | 1 |
| Q 21 | 1 | 0 | 1 | UTD | 1 | 1 | 1 | 1 | 1 | 1 | 1 |
| Q 22 | 1 | 1 | 1 | 1 | 1 | 1 | 1 | UTD | 1 | 1 | 1 |
| Q 23 | 0 | 0 | 0 | 0 | 0 | 0 | 0 | 0 | 0 | 0 | 0 |
| Q 24 | 0 | 0 | 0 | 0 | 0 | 0 | 0 | 0 | 0 | 0 | 0 |
| Q 25 | 1 | 1 | 1 | 0 | 1 | 1 | 1 | 0 | 1 | 0 | 1 |
| Q 26 | 0 | 0 | 0 | UTD | 0 | 0 | 0 | 0 | 1 | 0 | 0 |
| Q 27 | 1 | UTD | 1 | UTD | 1 | UTD | UTD | 1 | UTD | UTD | UTD |

## Supplementary Table 4: Risk of bias for randomized control trials included in the systematic review

| **Entry** | **Lazarus** | **Casey** |
| --- | --- | --- |
| Random Sequence Generation | Low Risk | Low Risk |
| Allocation Concealment | Low Risk | Low Risk |
| Blinding of Participants and Personnel | High Risk | Low Risk |
| Blinding of outcome assessment (patient-report) | Low Risk | Low Risk |
| Blinding of outcome assessment (mortality) | Low Risk | Low Risk |
| Incomplete Outcome data assessed (Short term) | High Risk | Low Risk |
| Incomplete Outcome data assessed (Long term) | Unclear Risk | Low Risk |
| Selective Reporting | Low Risk | Low Risk |

**Supplementary Table 5:** Observational studies of subclinical hypothyroidism and hypothyroxinaemia association with offspring intellectual disability included and excluded from meta-analysis (with reasons for exclusion). Two studies (Andersen et al^37^ and George et al^50^) on association of autism with overt hypothyroidism based on hospital records were included in the meta-analysis. Two studies (Román et al^3^ and Brown et al^40^) on association of autism with hypothyroxinaemia and subclinical hypothyroidism respectively were not included in the meta-analysis as there were no other studies to meta-analyse them with in their respective categories. Both randomised controlled trials (Lazarus 2012^7^ and Casey 2017^49^) were included in the meta-analysis.

| **Study** | **Subclinical Hypothyroidism** | **Hypothyroxinaemia** |
| --- | --- | --- |
| Pop, 1999 | Excluded (only measured psychomotor outcomes) | Excluded (only measured psychomotor outcomes) |
| Haddow, 1999/Klein, 2001 | Included | Excluded (no data on hypothyroxinaemia) |
| Smit, 2000 | Included | Excluded (no data on hypothyroxinaemia) |
| Pop, 2003 | Excluded (no data on subclinical hypothyroidism) | Included |
| Kasatkina, 2006 | Excluded (no data on subclinical hypothyroidism) | Included |
| Kooistra, 2006 | Excluded (only measured psychomotor outcomes) | Excluded (only measured psychomotor outcomes) |
| Oken, 2009 | Excluded (linear regression results that didn’t give unadjusted value) | Excluded (used continuous predictor) |
| Munoz, 2009 | Excluded (no data on subclinical hypothyroidism) | Included |
| Henrichs, 2010 | Excluded (used continuous predictor) | Included |
| Li, 2010 | Included | Included |
| Ghassabian, 2011 | Excluded (used continuous predictor) | Excluded (used continuous predictor) |
| Su, 2011 | Included | Excluded (no data on hypothyroxinaemia) |
| Chevrier, 2011 | Excluded (used continuous predictor) | Excluded (used continuous predictor) |
| Behrooz, 2011 | Included | Excluded (no data on hypothyroxinaemia) |
| Williams, 2012 | Included | Included |
| Suárez-Rodríguez, 2012 | Excluded (no data on subclinical hypothyroidism) | Included |
| Craig, 2012 | Excluded (no data on subclinical hypothyroidism) | Included |
| Williams, 2013 | Included | Excluded (no data on hypothyroxinaemia) |
| Julvez, 2013 | Excluded (linear regression results that didn’t give unadjusted value) | Excluded (linear regression results that didn’t give unadjusted value) |
| Finken, 2013 | Excluded (used novel and unvalidated outcome assessment) | Excluded (used novel and unvalidated outcome assessment) |
| Roman, 2013 | Excluded (used continuous predictor) | Excluded (no other studies to meta-analyse with) |
| Andersen, 2013 | Excluded (no data on this type of hypothyroidism) | Excluded (no data on hypothyroxinaemia) |
| Ghassabian, 2014/ Korevaar, 2016 | Excluded (used continuous predictor) | Included |
| Yau, 2014 | Excluded (used continuous predictor) | Excluded (no data on hypothyroxinaemia) |
| George, 2014 | Excluded (no data on subclinical hypothyroidism) | Excluded (no data on hypothyroxinaemia) |
| Pakkila, 2014 | Included | Included |
| Korevaar, 2015 | Excluded (lacked useable numerical data for meta-analysis) | Excluded (lacked useable numerical data for meta-analysis) |
| Modesto, 2015 | Excluded (used continuous predictor) | Excluded (linear regression using log fT4, making it incomparable) |
| Grau, 2015 | Excluded (no data on subclinical hypothyroidism) | Included |
| Ganaie, 2015 | Excluded (only measured psychomotor outcomes) | Excluded (only measured psychomotor outcomes) |
| Pakkila, 2015 | Included | Included |
| Murphy, 2015 | Included | Excluded (no data on hypothyroxinaemia) |
| Chen, 2015 | Included | Excluded (no data on hypothyroxinaemia) |
| Noten, 2015 | Included | Included |
| Brown, 2015 | Excluded (no other papers to meta-analyse with) | Excluded (used a continuous predictor) |

## Supplementary Table 6: Sensitivity analysis of different methods for converting continuous results and regression coefficients into odds ratios for meta-analysis

| **Methods used** | **Outcome** | **Studies included** | **Trait measured** | **Odds ratio (95% CI)** |
| --- | --- | --- | --- | --- |
| Chinn method and odds ratios | Indicators of intellectual disability | Haddow 1999, Smit 2000, Li 2010, Su 2011, Behrooz 2011, Williams 2013, Chen 2015, Noten 2015, Williams 2012, Murphy 2015, Pakkila 2015 | Subclinical Hypothyroidism | 2.14 (1.20-3.83) |
| Suissa & Whitehead method and odds ratios | Indicators of intellectual disability | Haddow 1999, Smit 2000, Li 2010, Su 2011, Behrooz 2011, Williams 2013, Chen 2015, Noten 2015, Pakkila 2015 | Subclinical Hypothyroidism | 2.38 (1.10-5.15) |
| Odds ratios only | Indicators of intellectual disability | Haddow 1999, Li 2010, Su 2011, Chen 2015, Noten 2015, Pakkila 2015 | Subclinical Hypothyroidism | 2.37 (0.96-5.85) |
| Chinn method and odds ratios | Indicators of intellectual disability | Pop 2003, Kasatkina 2006, Suárez-Rodríguez 2012, Pakkila 2015, Grau 2015, Li 2010, Henrichs 2010, Ghassabian 2014, Noten 2015, Williams 2012, Craig 2012 | Hypothyroxinaemia | 1.63 (1.03-2.56) |
| Suissa & Whitehead method and odds ratios | Indicators of intellectual disability | Pop 2003, Kasatkina 2006, Suárez-Rodríguez 2012, Pakkila 2015, Grau 2015, Li 2010, Henrichs 2010, Ghassabian 2014, Noten 2015 | Hypothyroxinaemia | 2.02 (1.18 – 3.45) |
| Odds ratios only | Indicators of intellectual disability | Pakkila 2015, Li 2010, Henrichs 2010, Noten 2015 | Hypothyroxinaemia | 2.11 (0.92-4.83) |
